# Effect of Soybean Variety on Anti-Nutritional Factors Content, and Growth Performance and Nutrients Metabolism in Rat

**DOI:** 10.3390/ijms11031048

**Published:** 2010-03-09

**Authors:** Chunmei Gu, Hongbin Pan, Zewei Sun, Guixin Qin

**Affiliations:** 1 Institute of Food Science and Engineering, Jilin Agricultural University, Changchun, Jilin 130118, China; 2 Institute of Animal Science and Technology, Yunnan Agricultural University, Kunming, Yunnan Province 650201, China; 3 Institute of Animal Science and Technology, Jilin Agricultural University, Changchun, Jilin 130118, China

**Keywords:** soybean variety, anti-nutritional factors, rat, growth performance, nutrient digestibility

## Abstract

The present study was undertaken to evaluate the effects of soybean varieties on content of anti-nutritional factors, growth, and nutrient digestibility in rat. For this purpose, the content of trypsin inhibitor and lectin was firstly measured in five soybean varieties. Then sixty male Wistar rats were randomly divided into six groups and fed on different diets as follows: groups 1 to 5 were fed on treatment diets containing five different varieties of soybean flour; group 6 was fed on a control diet containing casein. All animals were fed for four weeks. During this period, faeces and urine were collected to determine the nutritional efficiency of diets and body weight were measured weekly on ten rats from each group. The results showed that trypsin inhibitor and lectin content of Jilin45 was the highest, and those of Jinong7 were the lowest of the soybean varieties. In comparison, all measured parameters, that is including gain in body weight, feed utilization efficiency, nutrient digestibility, nitrogen balance and nitrogen retention, were markedly different among the five groups of animals, but were significantly lower than the control group. These findings show that soybean varieties could significantly affect trypsin inhibitor and lectin content in soybean.

## 1. Introduction

As the main source of vegetable protein, soybean plays an important role in the world of animal feed and food production. It is a major source of both vegetable protein for humans and animals and of edible oil [1]. However, it contains many kinds of anti-nutritional factors, such as trypsin inhibitor, lectin, α-amylase inhibiting factor, goitrin, soybean antigen and so on [2,3]. The existence of these anti-nutritional factors affects the nutritional value, utilization and digestibility of soybean protein [4,5], causing digestive and metabolic diseases of animals [6,7] as well as reduced performance traits [8]. Consequently, soybean must be subjected to thermal treatment before consumption by human beings or animals, in order to destroy or reduce this anti-nutritional content, but the thermal treatment process could also destroy other essential nutrients, and it raises the cost and energy demands of soybean production. Despite the heat treatment of soybeans, findings have shown that it still contains low-levels of anti-nutritional factors, such as phytoagglutinin [9]. Therefore, the present study was designed to analyze and compare the trypsin inhibitor and lectin content in five important cultivated soybean varieties that are generally cultivated in Jilin province, and also to investigate the effect of anti-nutritional factors of different soybean varieties on animal metabolism. These results would provide a reference for soybean breeding, and reasonable processing methods, utilization and breeding of soybean varieties with low-levels of anti-nutritional factors.

## 2. Materials and Methods

### 2.1. Animals and Diets

Male Wistar rats, weighing 80 ± 5 g at the beginning of the experiment, were given a balanced control diet (200 g casein/kg) for 1 week. After this adaptation period their weight was 100 ± 8 g. They were then divided randomly into six equal groups of ten animals each and fed on different diets as follows: groups 1 to 5 were fed on a treatment diet containing five different varieties of soybean flour, (Jilin parvule 6, Jilin30, Jilin45, Jinong7, and Fengjiao7607) for four weeks; group 6, a control group, was fed on the original reference diet. Diets were isoenergetic (16.9 MJ/kg) and were given in powdered form. The composition of the diets is shown in Table 1. Rats were kept in wire-bottomed cages under a controlled atmosphere (temperature, 23 ± 1 °C; relative humidity, 55 ± 5%; and a fixed 12-h light: dark cycle, light 0700 to 1,900 h). They had *ad libitum* access to food and tap water. The care and use of laboratory animals followed the institutional guidelines of Jilin Agricultural University.

Food consumption was recorded daily. Body weight was measured weekly on ten rats from each group before feeding in the morning. To determine the nutritional efficiency of diets, urine and faeces were collected and pooled for nine days. This collection period was arbitrarily divided into two parts of four and five days, respectively. Analyses were carried out separately for the samples collected in these two periods and the results were combined and recorded only if there were no disproportionally large differences between them. Commercially available soybean flour was purchased from Jilin Province Agriculture Academy of Science (Jilin, China).

### 2.2. Analytical Methods

#### 2.2.1. Trypsin Inhibitor and Lectin Determination

The content of soybean trypsin inhibitor in soybean was measured by the procedure of Liu and Markakis [10]. In this procedure, trypsin inhibitor in samples reacts with trypsin for 2–3 minutes, then 30% acetic acid was added to terminate the reaction, and then inhibited trypsin was monitored spectrophotometrically at 410 nm. Lectin content in soybean was assayed according to the method of Pusztai and Grant [9] using an enzyme linked immunosorbent assay.

#### 2.2.2. Growth Performance Assay

Body weight and feed consumption were weighted in rats before feeding in the morning once a week, and body weight gain and feed conversion ratio (F:G) were calculated.

#### 2.2.3. Nutrients Digestibility Determination

The samples of experimental diets and faeces were homogenized using a motor and pestle and analyzed by standard methods (AOAC, 1995). The nutrient’s digestibility was measured by the method of Noreen and Salim [11]. The dry matter was determined by oven-drying at 105 °C for 16 h; crude protein (N × 6.25) by micro Kjeldahl apparatus; crude fat by petroleum ether extraction method; Nitrogen Free Extract (NFE) was calculated by taking the sum of values for crude protein, crude fat, ash, crude fiber and subtracting this from 100.

#### 2.2.4. Nitrogen Balance and Nitrogen Retention Assay

Intake nitrogen, fecal nitrogen and urinary nitrogen were determined by the method of Noreen and Salim [11], and nitrogen balance and nitrogen retention were calculated according to the following formulas:

$$\begin{array}{l} \text{Nitrogen}\;\text{balance}=\text{intake}\;\text{nitrogen}\;-\;\text{urinary}\;\text{nitrogen}\;-\;\text{fecal}\;\text{nitrogen}\\ \text{Nitrogen}\;\text{retention}=(\text{intake}\;\text{nitrogen}\;-\;\text{urinary}\;\text{nitrogen}\;-\;\text{fecal}\;\text{nitrogen})/\text{intake}\;\text{nitrogen} \end{array}$$

### 2.3. Statistical Analysis

Data Analysis: Data are reported as means ± SD, *n* = 10. Differences between mean values were determined by ANOVA followed by comparisons using the Newman-Keuls multiple range test. Differences with *P* < 0.05 were considered significant.

## 3. Results

### 3.1. Content of Trypsin Inhibitor and Lectin in Five Varieties of Soybean

Table 2 shows significant differences in the content of anti-nutritional factors among the five tested soybean varieties. The content of trypsin inhibitor in Jilin 45 (59.46 mg/g) was the highest compared with that of other varieties. That of Jinong 7 was the lowest and markedly different from the other three species (P < 0.05). For the content of lectin, that of Jilin 45(6.52 mg/g) was significantly higher than other varieties (P < 0.05), while Jilin parvule 6 (3.51 mg/g) was rated next to Jilin 45 and both were markedly different from the other varieties (P < 0.05).

### 3.2. The Effects of Soybean Varieties on Growth Performance in Rats

As a result of the presence of anti-nutritional factors the body-weight and feed conversion ratios were strikingly decreased (P < 0.05), as is evident from Table 3. Body-weight gain in Jilin45 group was the lowest, while that of Jinong7 group was the highest when compared with other treatment groups; feedstuff/gain in weight in Jilin45 group was the highest and that of Jinong7 group was the lowest, they were significantly different from other treatment groups (P < 0.05).

### 3.3. The Effect of Soybean Varieties on Nutrient Digestibility

The anti-nutritional factors lowered the nutrient digestibility of the experimental diets compared to control group, except for fat digestibility (Table 4). The digestibility of dry matter, protein and nitrogen free extract were the lowest in Jilin45 group and the highest in Jinong7 group, and there were significant differences between the values (P < 0.05). The presence of trypsin inhibitor and lectin resulted in a significant increase in fat digestibility, which was highest in the Jinong7 group and the lowest in the Jilin45 group, and significantly different between values (P < 0.05).

### 3.4. The Effect of Soybean Varieties on Nitrogenous Metabolism

Table 5 presents the effects of the anti-nutritional factor on nitrogen balance and nitrogen retention. The nitrogen balance and retention were significantly lower in treatment groups compared with the control group. The striking difference in these parameters in the five treatment groups was attributed to the different soybean varieties. Nitrogen balance and nitrogen retention among treatment groups were the highest in the Jinong7 group and the lowest in the Jilin45 group, and these were significantly different from other three treatment groups (P < 0.05).

## 4. Discussions

Qin *et al*. [12] reported that the trypsin inhibitor content in different soybean sources was different. Fu *et al*. [13] found that trypsin inhibitor content in L81-4387 was 16.5 units/g, but that in L81-45907 it was 17.6 units/g. In the present study, the results showed that soybean variety had a marked effect on the content of anti-nutritional factors like trypsin inhibitor and lectin. The content of anti-nutritional factors in Jilin 45 was markedly higher than in the other four tested soybean varieties (P < 0.05), and was lowest in Jinong7. These results may be attributed to genetic differences in soybean varieties, climate, environment, geographical factors, which could affect the content and activity of the soybean anti-nutritional factors.

Many studies have confirmed that daily gain in body weight and feed utilization efficiency are lower in animals fed with diets containing trypsin inhibitor and lectin [14–16]. In the present study, the body weight gain and feed utilization efficiency of rats fed with raw soybean diet were significantly lower, while those animals in the Jilin45 group had the lowest values. One of the possible reasons for this result is that trypsin inhibitor leads to the loss of endogenous nitrogen; the other is that lectin could combine with small intestine epithelium and induce constitutional and functional changes in the small intestine [17]. Liener [18] reported that 50% of growth inhibition in rat fed with raw soybean diet was attributed to lectin, 40% to trypsin inhibitor, and 10% to others. Therefore, the main anti-nutritional factors in soybean that inhibit growth in animals are trypsin inhibitor and lectin.

Schulze *et al*. [8] discovered that by adding 2.4 g/kg Kunitz Trypsin Inhibitor (KTI) to a control diet, the growth of animals was decreased by 13% and with the addition of 7.2 g/kg KTI, that was decreased by 32%. This indicates that the effect of soybean trypsin inhibitor on animal growth is related to its levels in diet. Grant *et al*. [19] also reported that the effect of lectin on growth performance of animals changed with its dosage. Li [20] observed that when the concentration of lectin in diet was between 0–1.2 mg/g, no obvious change of growth performance in rats was found in 20 days, but when above 2.0 mg/g, growth of rats was obviously lower, and decreased by 23% compared with control rats. In the present study, body weight gain and feed utilization efficiency with a diet of Jilin45 containing high levels of trypsin inhibitor and lectin were the lowest, while those with a diet of Jinong7 containing low levels of anti-nutritional factors were the highest. This result shows that the higher the anti-nutritional factor content is, the lower the body weight gain and feed utilization efficiency are, and this further demonstrates that soybean varieties have an obvious effect on the content of trypsin inhibitor and lectin, and lead to significant differences in body weight gain and feed conversion ratio in the five treatment groups.

Digestion and absorption of nutrients could be measured by two parameters, including nutrient digestibility and deposit. In the present study, raw soybean led to a significant decrease in dry matter, protein and nitrogen free extract digestibility. In previous studies, similar results were reported [8,21]. Nutrient digestibility was decreased in animals fed with trypsin inhibitor, for example pig, chicken, rat and mouse and so on, and the decrease of protein digestibility was pronounced the biggest. Many studies have found that protein digestibility was decreased by 20–40% in animals fed with diets containing raw soybean or high levels of trypsin inhibitor compared with those fed with diets containing heated soybean or soybean meal [22–24]. In the present study, raw soybean was used as the source of trypsin inhibitor, and the results showed that protein digestibility was significantly decreased by 22.56% in the Jilin45 treatment group containing the highest level of trypsin inhibitor compared with the control group. Qin [25] reported that lectin could combine with a specific receptor (polyose) of the epithelial cell surface in the small intestine wall, destroying the brush border mucosa structure of the small intestine, interfering with the function of many enzymes in the brush border mucosa, so as to decrease of protein utilization efficiency. In the present study, the results showed that protein digestibility was lowered in rats fed with lectin. In addition, the present study discovered that fat digestibility was significantly higher in treatment groups compared with control group, which was possibly attributed to a longer retention time of chymus in digestive tract and thorough digestion of fat.

Many studies have demonstrated that the effect of lectin on nitrogen metabolism was obvious, and it mainly increases the effluence of endogenous nitrogen so as to decrease nitrogen balance and nitrogen retention [26–28]. Li [20] discovered that with increase of lectin content in diet, the nitrogen loss showed a linear increase, but the nitrogen balance and nitrogen retention showed a linear decrease. In addition, the level of trypsin inhibitor also affects these two parameters. Hagemeister and Barth [29] reported that with an increase of trypsin inhibitor content in the diet, endogenous nitrogen in chymus was obviously increased, and nitrogen balance and nitrogen retention were obviously decreased. These results indicate that trypsin inhibitor and lectin could lower nitrogen deposit, thereby affecting digestion and absorption of the nutrient. In the present study, similar results were obtained. Trypsin inhibitor and lectin led to a significant decrease of nitrogen balance and nitrogen retention in treatment groups compared to those of control group; the extent of the decrease in the Jilin45 group was the highest, while that of the Jinong7 group was the smallest. These data show that the higher the trypsin inhibitor and lectin content in soybean is, the lower the nutrient digestibility, nitrogen balance and nitrogen retention are. On the basis of above results, it could be concluded that different soybean varieties have different anti-nutritional factors contents. Therefore, the study of the differences in anti-nutritional factor content in different soybean varieties and their effects on animals have important significance.

## Figures and Tables

**Table 1.** Composition of the diets (g/kg)^a^.

	**Ingredients**	**Control diet**	**Soybean flour**
Protein	Casein^b^	200	-
	Soybean protein^c^	-	200
Carbohydrates	Maize starch^d^	560	435
	Soybean carbohydrates^c^	-	125
Fat	Soybean oil^d^	120	15
	Soybean fat^c^	-	105
Fibres	Cellulose	60	42.5
	Soybean fibre^c^	-	17.5
Others	Salt mix^e^	40	40
	Vitamin mix^f^	20	20

a Diets were semipurified, isoenergetic (16.9 MJ/kg) and were given in powdered form.

b Shanghai, China.

c Ingredients originating from the soybean flour.

d Changchun, China.

e The salt mixture provides the following amounts (g/kg diet^−1^): Ca, 4; K, 2.4; Na, 1.6; Mg, 0.4; Fe, 0.12; trace elements: Mn, 0.032; Cu, 0.005; Zn, 0.018; Co, 0.00004; I, 0.00002.

f The vitamin mixture provides the following amounts (mg/kg diet^−1^): retinol, 12; cholecalciferol, 0.125; thiamin, 40; riboflavin, 30; pantothenic acid, 140; pyridoxine, 20; inositol, 300; cyanocobalamine, 0.1; ascorbic acid, 1600; (dL) α-tocopherol, 340; menadione, 80; nicotinic acid, 200; paraaminobenzoic acid, 100; folic acid, 10; biotin, 0.6; choline, 2720.

**Table 2.** Soybean trypsin inhibitor and lectin content(mg/g).

**Soybean variety**	**Trypsin inhibitor**	**Lectin**
Jilin parvule6	37.22 ± 0.25^B^	3.51 ± 0.03^B^
Jilin30	38.67 ± 0.36^B^	2.89 ± 0.01^A^
Jilin45	59.46 ± 1.51^C^	6.52 ± 0.02^C^
Jinong7	28.61 ± 0.43^A^	2.81 ± 0.01^A^
Fengjiao7607	38.47 ± 0.30^B^	2.84 ± 0.01^A^

Values are means ± SD, *n* = 10. Within an array, values without a common superscript are significantly different, *P* < 0.05.

**Table 3.** Body-weight gain of rat and feed conversion ratio.

**Groups**	**Body-weight gain(g)**	**Feedstuff/gain in weight**
Control diet	93.94 ± 0.03^D^	4.82 ± 0.02^A^

Jilin parvule 6	77.37 ± 0.95^B^	6.22 ± 0.01^C^
Jilin 30	77.00 ± 0.06^B^	6.25 ± 0.01^C^
Jilin45	69.70 ± 0.66^A^	8.11 ± 0.01^D^
Jinong7	87.75 ± 0.73^C^	5.42 ± 0.01^B^
Fengjiao7607	78.39 ± 0.12^B^	6.23 ± 0.02^C^

Values are means ± SD, n = 10. Within an array, values without a common superscript are significantly differ, P < 0.05.

**Table 4.** Main nutrient digestibility of diet (%).

**Groups**	**Dry matter**	**Protein**	**Fat**	**Nitrogen free extract**
Control diet	89.90 ± 0.61^D^	80.33 ± 0.20^E^	83.17 ± 0.15^A^	91.35 ± 0.43^D^

Jilin parvule6	79.03 ± 0.21^B^	66.45 ± 0.28^C^	87.87 ± 0.12^C^	88.29 ± 0.23^B^
Jilin 30	77.97 ± 0.32^B^	65.27 ± 0.26^B^	87.57 ± 0.07^C^	88.65 ± 0.07^B^
Jilin45	70.49 ± 0.33^A^	62.21 ± 0.28^A^	85.88 ± 0.13^B^	87.31 ± 0.22^A^
Jinong7	82.81 ± 0.69^C^	72.29 ± 0.61^D^	89.61 ± 0.25^D^	89.81 ± 0.19^C^
Fengjiao7607	78.29 ± 0.43^B^	65.00 ± 0.02^B^	87.36 ± 0.19^C^	88.15 ± 0.15^B^

Values are means ± SD, n = 10. Within an array, values without a common superscript are significantly differ, P < 0.05.

**Table 5.** Nitrogen balance and nitrogen retention.

**Groups**	**Nitrogen balance**	**Nitrogen retention**
Control diet	0.99 ± 0.02^D^	0.88 ± 0.01^D^

Jilin parvule 6	0.81 ± 0.01^B^	0.71 ± 0.01^B^
Jilin 30	0.82 ± 0.01^B^	0.72 ± 0.02^B^
Jilin45	0.70 ± 0.02^A^	0.62 ± 0.01^A^
Jinong7	0.90 ± 0.01^C^	0.78 ± 0.01^C^
Fengjiao7607	0.83 ± 0.01^B^	0.72 ± 0.01^B^

Values are means ± SD, n = 10. Within an array, values without a common superscript are significantly differ, P < 0.05.
